# TIF1 Family Proteins as Modulators of Cell Death: Mechanisms and Therapeutic Opportunities

**DOI:** 10.3390/biom16050719

**Published:** 2026-05-13

**Authors:** Dong Yang, Yuchen Chen

**Affiliations:** 1 Division of Nephrology, Department of Medicine, University of Connecticut School of Medicine, Farmington, CT 06030, USA; doyang@uchc.edu; 2 Division of Surgical Sciences, Department of Surgery, University of Virginia, Charlottesville, VA 22903, USA

**Keywords:** TRIM24, TRIM28, TRIM33, TRIM66, cell death, apoptosis, ferroptosis, pyroptosis, necroptosis, therapeutic targeting

## Abstract

Regulated cell death is essential for development, tissue homeostasis, host defense, and disease. Beyond apoptosis, it is now clear that other forms of cell death, including ferroptosis, pyroptosis, and necroptosis, also contribute to pathology, often in interconnected rather than isolated ways. Within this broader framework, the transcriptional intermediary factor 1 (TIF1) family, comprising TRIM24, TRIM28, TRIM33, and TRIM66, has emerged as an important group of regulators linking stress adaptation, cell-state control, and cell death susceptibility. Although these proteins belong to the same family, they influence cell death through distinct and context-dependent mechanisms. Across the TIF1 family, apoptosis is by far the most extensively studied cell death phenotype, whereas links to ferroptosis, pyroptosis, and necroptosis remain more limited, more context dependent, and more unevenly distributed across individual members. Cell death often becomes evident when TIF1-dependent stress-buffering programs are disrupted, highlighting both their biological importance and potential therapeutic relevance. At the same time, family-level differences are emerging, while the underlying mechanisms remain incompletely understood, and recent advances in this field have not been synthesized. This review summarizes how TIF1 family members intersect with different cell death programs, discusses emerging translational opportunities and challenges, and highlights key mechanistic questions for future study.

## 1. Introduction

Regulated cell death is fundamental to development, tissue homeostasis, host defense, and disease pathogenesis [1,2]. While apoptosis has long been the most extensively studied form of cell death, the field now recognizes multiple additional forms, including ferroptosis, an iron- and lipid peroxidation-driven form of cell death [3,4]; pyroptosis, a gasdermin-mediated inflammatory death program [5]; necroptosis, a lytic pathway typically driven by receptor-interacting serine/threonine-protein kinase 3 (RIPK3) and mixed lineage kinase domain-like protein (MLKL) [6]; and autophagy-dependent cell death, in which the autophagy machinery directly contributes to cell death [7]. Importantly, these forms are not isolated modules. Accumulating evidence indicates that cell death is part of an interconnected signaling network, in which shared stress signals, molecular checkpoints, and compensatory mechanisms influence whether and how cells die [8,9]. This conceptual shift is illustrated by PANoptosis, an integrated inflammatory cell death program that brings together components of pyroptosis, apoptosis, and necroptosis rather than activating them as fully independent pathways. More broadly, regulated cell death is increasingly understood as a downstream output of stress-response systems shaped by chromatin state, inflammatory signaling, metabolic rewiring, organelle quality control, and genome surveillance [10,11]. This has drawn increasing attention to upstream regulators that do not merely execute cell death, but instead shape death susceptibility, stress adaptation, and pathway choice across contexts.

Within this landscape, the transcriptional intermediary factor 1 (TIF1) family—TRIM24 (TIF1α), TRIM28 (TIF1β), TRIM33 (TIF1γ), and TRIM66 (TIF1δ)—stands out as a particularly interesting group [12,13]. These proteins form a distinctive subgroup within the tripartite motif (TRIM) superfamily. TIF1 family members generally share an N-terminal TRIM/RBCC module (RING, B-box, and coiled-coil domains) linked to protein assembly and ubiquitin-related signaling, together with a C-terminal plant homeodomain (PHD)-bromodomain cassette that mediates chromatin regulation and transcriptional control; TRIM66 is often considered the structural outlier because it lacks a canonical RING domain [14,15]. This modular organization suggests that TIF1 proteins are not merely broad transcriptional coregulators recruited to promoters or enhancers. Instead, their RBCC–PHD–bromodomain architecture enables them to integrate ubiquitin/SUMO-related post-translational modification (PTM) signaling with chromatin-state recognition, thereby influencing cell fate through two interconnected mechanisms: chromatin-dependent transcriptional regulation and PTM-dependent control of substrate stability or signaling complex assembly. Together, these mechanisms may determine whether cells undergo regulated cell death [12].

Despite their shared module organization, TIF1 family members are not functionally redundant in cell death regulation. Current evidence suggests that different family members intersect with cell death pathways in distinct ways. In recent years, TRIM24 and TRIM28 have also drawn translational interest [16,17]. TRIM24 is especially attractive from a drug-development perspective because its bromodomain provides a tractable entry point for small-molecule targeting [18,19], whereas TRIM28 continues to attract attention because of its broad upregulation across cancers and its links to prognosis and treatment resistance [17]. However, the literature remains scattered across multiple fields and is usually considered in a protein-specific or disease-specific context. A family-level review is therefore needed to bring out shared patterns and knowledge gaps, clarify member-specific roles, and highlight potential translational opportunities.

In this review, we summarize how TRIM24, TRIM28, TRIM33, and TRIM66 influence different cell death programs across cancer, development, inflammation, degeneration, and therapy response. We also highlight kidney injury and kidney disease as emerging contexts in which TIF1 family proteins may shape stress responses, maladaptive repair, and cell death susceptibility. In addition, we discuss translational implications, with a focus on representative efforts to target TIF1 proteins pharmacologically. Finally, we compare shared versus member-specific characteristics across the family and outline key directions for future research.

## 2. A Chromatin–PTM Coupling Model for TIF1-Mediated Death-Threshold Regulation

Existing studies have established TIF1 family proteins as modular TRIM cofactors that connect chromatin regulation, transcriptional control, genome stability, and stress signaling [13,20,21,22,23]. However, their roles in regulated cell death require a more focused structure-function framework. In this review, we use the term “modulator” to describe proteins that alter the susceptibility of cell death responses, rather than proteins that directly constitute the core execution machinery of a specific death pathway. From this perspective, TIF1 proteins are best understood as regulators of cell death thresholds rather than canonical death executioners. We therefore propose a “chromatin–PTM coupling model” for TIF1-mediated death-threshold regulation.

### 2.1. Shared but Non-Identical Domain Architecture of TIF1 Family Proteins

TRIM24, TRIM28, and TRIM33 belong to the PHD–bromodomain-containing C-VI subgroup of TRIM proteins and generally contain an N-terminal TRIM/RBCC module linked to a C-terminal PHD–bromodomain cassette [13]. The RBCC region supports oligomerization, scaffold formation, substrate recruitment, and ubiquitin/SUMO-related PTM signaling, whereas the PHD–bromodomain cassette functions as a chromatin-reading module that recognizes histone modification states to control gene transcription [24]. For example, TRIM24’s PHD fingers can read the methylation state of H3K4, while bromodomains recognize acetylated lysine [25]. Notably, all four TIF1 proteins contain a conserved TIF1 signature sequence (TSS) implicated in transcriptional repression [16]. Additionally, TRIM24, TRIM28, and TRIM66 contain heterochromatin protein 1 (HP1)-binding domains that interact with HP1 family proteins through a PxVxL motif [16,26].

TRIM66 is structurally more divergent: although it shares the C-terminal PHD–bromodomain cassette, it lacks the same canonical RING-containing RBCC architecture seen in TRIM24, TRIM28, and TRIM33. This distinction is important because TRIM66 is better viewed as a chromatin-state reader and genome-stress regulator than as a classical RING-dependent E3 ligase. Consistent with this view, structural and functional studies show that the TRIM66 PHD–bromodomain recognizes unmodified H3R2/H3K4 and H3K56ac and supports DNA damage responses and genome stability [23].

### 2.2. PTMs of TIF1 Proteins in Association with Stress Response

Post-translational modifications on TIF1 proteins provide an important regulatory layer that controls their stability, chromatin association, scaffold function, and stress-responsive activity. Among the four family members, TRIM28 has the best-defined PTM regulatory landscape. TRIM28 phosphorylation at S473 and S824—mediated by ataxia-telangiectasia mutated protein (ATM) [27,28], checkpoint kinase 1/2 (Chk1/Chk2) [29], p38 mitogen-activated protein kinase (p38 MAPK) [30], DNA-dependent protein kinase (DNA-PK) [31], NIMA-related kinase 9 (NEK9) [32], or mechanistic target of rapamycin complex 1 (mTORC1) [33] in different contexts—regulates chromatin relaxation, DNA damage responses, E2F1 interaction, transcriptional repression, and stress-associated survival. TRIM28 SUMOylation at residues including K554, K779, and K804 supports transcriptional repression [34], whereas sentrin-specific protease 7 (SENP7)-mediated deSUMOylation promotes chromatin relaxation during DNA damage responses [35]. TRIM28 can also be regulated by ubiquitination, deacetylation, and methylation, which affect its abundance or scaffold function [36,37,38].

TRIM24 is also regulated by PTM-dependent stability and damage-response mechanisms, although its PTM map is less comprehensive than TRIM28. Speckle-type POZ protein (SPOP) mutations impair TRIM24 ubiquitination and degradation in prostate cancer. TRIM28 can protect TRIM24 from SPOP-mediated degradation [39], and DNA damage-associated ATM signaling promotes TRIM24 recruitment to damage sites, followed by self-ubiquitination and turnover [21]. In contrast, PTM maps for TRIM33 and TRIM66 remain less complete.

Compared with domain organization and self-PTM regulation, mutation-level evidence directly connecting specific TIF1 variants to defined cell death outcomes remains limited. Available mutation or alteration data more often support changes in protein stability, chromatin binding, genome stability, or tumor behavior rather than direct mutation-driven control of specific cell death modality.

### 2.3. Implications for Cell Death Regulation

Taken together, TIF1 proteins should not be viewed simply as broad transcriptional coregulators recruited to promoters or enhancers. Instead, they are positioned to integrate stress signals through two interconnected layers. One layer is nuclear and chromatin-associated, involving histone-mark recognition, transcriptional regulation, heterochromatin organization, and DNA damage responses [13]. The other layer is PTM- and substrate-centered, involving ubiquitin/SUMO-dependent control of protein stability, localization, autophagy regulation, inflammatory signaling complexes, and metabolic stress pathways [39,40,41]. These two layers are not independent: PTM-dependent regulation of transcription factors, chromatin modifiers, or signaling adaptors can reshape transcriptional programs, while chromatin state can determine which stress-response genes or substrate networks are available for activation.

During cell death stress, including DNA damage, oxidative stress, inflammatory stimulation, proteotoxic stress, or metabolic stress, these nuclear and non-chromatin mechanisms jointly influence whether cells repair damage, adapt, enter senescence, or cross the threshold into apoptosis, ferroptosis, pyroptosis, necroptosis, or other forms of regulated cell death [1]. Importantly, this chromatin–PTM coupling model does not imply that all TIF1 proteins regulate cell death through the same route. Depending on cell type, stress intensity, chromatin state, substrate availability, PTM linkage type, and pathway priming, the same TIF1 member may either suppress or promote a given death modality [42,43,44]. Therefore, the following sections focus specifically on TIF1-associated mechanisms that include cell death related readouts. We discuss TRIM24, TRIM28, TRIM33, and TRIM66 within a common framework, while highlighting the specific substrates, stress contexts, and cell death related readouts that distinguish each family member.

## 3. TRIM24 and Cell Death Programs

TRIM24 (TIF1α) is recognized as a modulator of cell fate decisions across cancer and inflammatory/degenerative diseases. Rather than acting as a single “on/off” switch, current studies suggest that TRIM24 shapes susceptibility to multiple death modalities in a context-dependent manner. Below, we summarize evidence linking TRIM24 to apoptosis, ferroptosis, inflammasome-driven pyroptosis, autophagy programs, and necroptosis-associated signaling (Table 1).

### 3.1. TRIM24 in Apoptosis and Genotoxic Stress Responses

**Figure 1:**
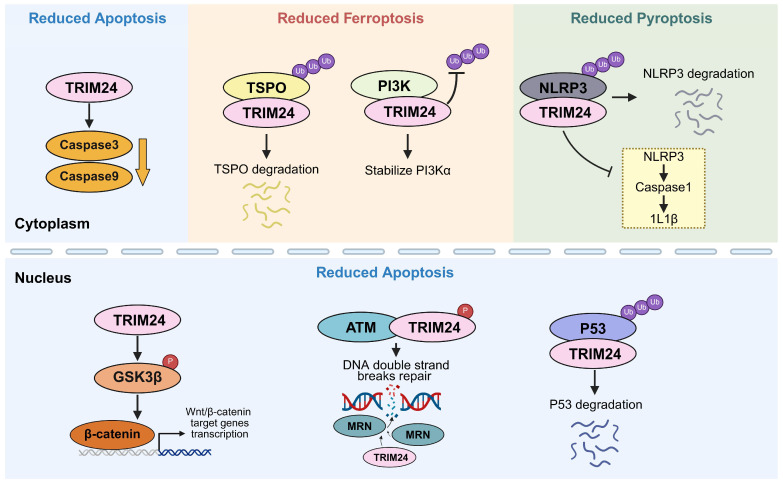
Representative mechanisms by which TRIM24 regulates apoptosis, ferroptosis, and pyroptosis. This schematic illustrates several known mechanisms by which TRIM24 modulates distinct cell death programs across different disease settings. Ub, ubiquitin. P, phosphorylation. Created in BioRender. Yang, D. (2026) https://BioRender.com/krvzjjx (accessed on 10 May 2026).

Evidence linking TRIM24 to cell death has been most extensively developed in tumor settings, where multiple studies report an overall pro-survival, anti-apoptotic effect for TRIM24 (Figure 1). Across several cancer types, elevated TRIM24 is associated with reduced apoptosis and more aggressive malignant phenotypes, whereas TRIM24 depletion induces apoptosis [49,50,51]. One mechanistic anchor for this pattern is that TRIM24 can ubiquitinate p53 and promote its downregulation, thereby blunting p53-driven apoptotic and tumor-suppressive responses [42], although the precise linkage type and substrate residue involved in this TRIM24-p53 axis remain incompletely defined. In nasopharyngeal carcinoma models, TRIM24 knockdown increased apoptosis, accompanied by upregulation of caspase-3/9 [52], and TRIM24 silencing similarly enhanced caspase-3/7 activity in clear cell renal cell carcinoma cells [53]. Likewise, TRIM24 depletion suppressed tumor growth and promoted apoptosis in acute myeloid leukemia, at least in part through downregulation of Wnt/glycogen synthase kinase-3 beta (GSK3β)/β-catenin signaling [48].

In addition to basal survival signaling, TRIM24 can also shape apoptotic responses to genotoxic therapy. In hepatocellular carcinoma, ATM-phosphorylated TRIM24 was recruited to DNA double-strand breaks and promoted MRN complex loading on chromatin; accordingly, TRIM24 depletion increased chemotherapy-induced apoptosis and slowed tumor growth in vivo [21].

### 3.2. TRIM24 in Ferroptosis

Beyond apoptosis, TRIM24 has also been reported to modulate ferroptosis vulnerability in cancer (Figure 1). In nuclear factor erythroid 2-related factor 2 (NRF2)-active lung squamous cell carcinoma (LUSC), TRIM24 expression increased upon NRF2 depletion, and loss of TRIM24 selectively promoted oxidative stress-associated ferroptosis alongside apoptosis. Mechanistically, TRIM24 depletion reduced TRIM24-phosphoinositide 3-kinase α (PI3Kα) complex formation and destabilized the PI3Kα catalytic subunit, thereby lowering the oxidative stress tolerance of NRF2-active LUSC cells [45]. Another study in colorectal cancer identified Oroxin A as a TRIM24 inhibitor that lowers TRIM24 protein levels and promotes ferroptosis, which may involve angiogenesis regulator translocator protein (TSPO). Mechanistically, TRIM24 downregulation decreased TSPO ubiquitination and increased TSPO abundance, inducing ferroptosis and inhibiting tumor development [46].

### 3.3. TRIM24 in Inflammasome-Associated Pyroptosis

Outside of cancer, recent studies extend TRIM24’s function to a negative regulator of NACHT, LRR, and PYD domains-containing protein 3 (NLRP3) inflammasome-driven pyroptosis (Figure 1). In endometriosis, TRIM24 expression was reduced, and TRIM24 deficiency enhanced NLRP3/caspase-1/interleukin-1 beta (IL-1β)-associated pyroptosis; mechanistically, TRIM24 interacted with NLRP3 and promoted its ubiquitination, thereby limiting inflammasome activation [47]. Consistently, in osteoarthritis, baicalin was reported to bind TRIM24 and increase TRIM24 protein levels, with TRIM24 in turn binding with NLRP3 and suppressing NLRP3/caspase-1-mediated chondrocyte pyroptosis [54]. Together, these findings support the view that TRIM24 can restrain inflammasome-driven pyroptotic signaling in inflammatory microenvironments, although key mechanistic details, such as the key interaction sites and patterns, remain to be established.

### 3.4. TRIM24 in Other Cell Death Modalities

Current knowledge about TRIM24’s impact on additional cell death modalities remains limited. Available studies suggest that TRIM24 rewires autophagy/proteostasis programs to support survival under stress. In bortezomib (BTZ)-resistant mantle cell lymphoma, TRIM24 was proposed to promote proteaphagy by favoring K63-linked ubiquitin chains, thereby sustaining cell survival and reducing BTZ sensitivity; notably, pharmacologic targeting of TRIM24 (dTRIM24) restored BTZ sensitivity and increased cell death in resistant cells [55]. In SPOP-mutant prostate cancer, loss of SPOP-dependent TRIM24 turnover leads to TRIM24 accumulation. Accumulated TRIM24 interacts with unc-51-like autophagy activating kinase 1 (ULK1) and promotes K27-linked ubiquitination of ULK1, thereby stabilizing ULK1, enhancing energy stress-induced autophagy, and supporting tumor cell stress adaptation and growth [39]. In addition, TRIM24 knockdown increased RIPK3 expression and accelerated disease features in osteoarthritis [56], suggesting a possible role for TRIM24 in restraining RIPK3-associated, necroptosis-related inflammatory injury, although its impact on canonical necroptotic execution remains to be explored.

TRIM24 repeatedly touches nodes that sit at the interface of inflammatory and stress-induced death programs. To date, TRIM24 has been linked in separate contexts to apoptotic susceptibility, NLRP3 inflammasome-driven pyroptosis, and RIPK3-associated necroptosis-related signaling. Given the growing interest in PANoptosis as an integrated inflammatory cell death program, these observations raise an interesting but currently unproven possibility that TRIM24 may influence PANoptosis-relevant pathway priming. At this stage, these findings should not be equated with bona fide PANoptosis, which requires coordinated activation of pyroptotic, apoptotic, and necroptotic pathways through PANoptosome complexes [57,58]. Currently, there is no direct evidence that TRIM24 physically associates with PANoptosome components or controls PANoptosome assembly, pathway switching, or coordinated death-modality activation. Nevertheless, because TRIM24 has been linked to apoptotic, inflammasome-related, and RIPK3-associated signaling nodes in separate contexts, this remains a worthwhile direction to test under defined inflammatory or stress conditions. Future studies using time-resolved, genetic, and biochemical approaches will be needed to determine whether TRIM24 participates in integrated death-pathway regulation or primarily alters the sensitivity of individual cell death pathways.

## 4. TRIM28 and Cell Death Programs

TRIM28 (TIF1β) is the earliest and most extensively studied member of the TIF1 family (Table 2). TRIM28 can mediate both epigenetic regulation and post-translational control. Accumulating studies suggest that TRIM28 tunes the threshold for apoptosis, ferroptosis, pyroptosis, as well as mitophagy/autophagy-dependent stress adaptation.

**Table 2:** Representative studies supporting TRIM28 involvement in apoptosis regulation.

Disease/Model	Expression	Impact on Cell Death	Mechanism	Ref
Osteosarcoma cell line	N/A	Suppress apoptosis	TRIM28 promoted p53 ubiquitination and degradation by interacting with MDM2, thereby inhibiting p53-HDAC1 complex formation and p53 acetylation.	[59]
Melanoma cell lines	N/A	Suppress apoptosis	Interaction between MAGE protein and TRIM28 promoted TRIM28/p53 complex formation and enhanced p53 suppression.	[60]
Human cancer cell lines	N/A	Suppress apoptosis	TRIM28 interacted with E2F1 and inhibited E2F1 acetylation by promoting E2F1-HDAC1 complex formation.	[61]
Tumor malignancy	N/A	Suppress apoptosis	TRIM28 ubiquitylated and destabilized XAF1.	[62]
HeLa cells and HEK293T cells	N/A	Suppress apoptosis	TRIM28 promoted SUMOylation of G3BP1, and G3BP1 SUMOylation modulated stress granule dynamics and suppressed ROS levels.	[63]
Myocardial I/R injury	Elevated	Increase apoptosis	TRIM28 ubiquitylated and destabilized GPX1, resulting in ROS accumulation.	[64]
Neural stem cells after I/R	Decreased p-TRIM28 (Ser824)	pTRIM28 Ser824 Suppress apoptosis	Phosphorylation of TRIM28 at Ser824 promoted its binding to PCNA and stabilized PCNA by inhibiting CUL4A-mediated binding and ubiquitination.	[65]
HeLa cells /Radiation	Elevated p-TRIM28 (Ser473)	pTRIM28 Ser473 suppresses apoptosis	Phosphorylation of TRIM28 at Ser473 was induced by DNA damage and was involved in TRIM28-HP1 and TRIM28-E2F1 complex formation. The S473A TRIM28 mutant lost the ability to inhibit E2F1-induced apoptosis.	[29]
Erythroid cells	N/A	Suppress apoptosis	TRIM28 regulated the mRNA expression of erythroid transcription factors, heme biosynthetic enzymes, and components of the apoptotic machinery. Genetic loss of TRIM28 in mice led to anemia.	[66]
Hematopoietic stem cells (HSCs)	N/A	Suppress apoptosis	TRIM28-HP1 complex maintained HSC-specific transcription. Deletion of TRIM28 in mice accelerated HSC apoptosis and efflux, leading to HSC depletion.	[67]
NSCLC	Elevated	Suppress apoptosis	TRIM28 affected the BCL2/BAX balance by upregulating BCL2 and suppressing BAX.	[68]
DDP-resistant A549 cells	Elevated	Suppress apoptosis	TRIM28 promoted miR-125b-5p expression, and miR-125b-5p, in turn, inhibited CREB1 expression in DDP-resistant cells.	[69]

### 4.1. TRIM28 in Apoptosis: Protein-Level and Transcriptional Regulation

**Figure 2:**
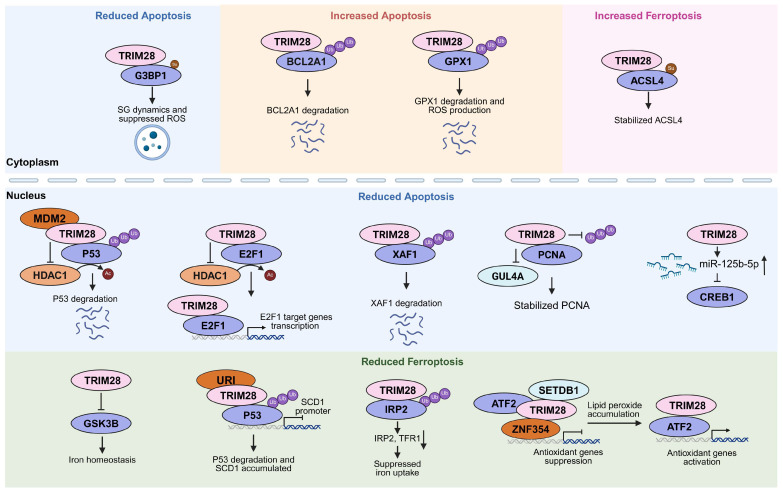
Representative mechanisms by which TRIM28 regulates apoptosis and ferroptosis. This schematic illustrates several known mechanisms by which TRIM28 modulates distinct cell death programs in different biological contexts. Ub, ubiquitin. Ac, acetylation. Su, SUMOylation. Created in BioRender. Yang, D. (2026) https://BioRender.com/6lq1i17 (accessed on 10 May 2026).

TRIM28’s role in cell death regulation is most extensively characterized in cancer, where TRIM28 frequently plays an anti-apoptotic role (Figure 2). TRIM28 expression is frequently increased across tumor types and has been linked to tumor initiation, progression, and drug response [68,70,71,72]. Mechanistically, these effects involve both protein-level control of apoptotic regulators and transcriptional regulation of pro-apoptotic gene programs (Table 2).

At the protein level, TRIM28 can interact with mouse double minute 2 (MDM2) to promote p53 deacetylation and ubiquitin-dependent downregulation, thereby dampening p53-driven apoptosis; accordingly, TRIM28 depletion sensitized cells to DNA damage-induced apoptosis [59]. In parallel, TRIM28 can limit E2F1-dependent apoptosis by facilitating E2F1-histone deacetylase1 (HDAC1) complex formation and inhibiting E2F1 acetylation [61], with TRIM28 S473 phosphorylation crucial for effective TRIM28-E2F1 interaction and anti-apoptotic function [29]. In addition, in multiple tumor cell lines, TRIM28 destabilized the pro-apoptotic protein X-linked inhibitor of apoptosis-associated factor 1 (XAF1) through K48-linked ubiquitination [62]. These examples illustrate how TRIM28 can suppress apoptosis by modifying the stability, acetylation status, or interaction networks of apoptotic regulators.

At the transcriptional level, knockdown of TRIM28 increased apoptosis and upregulated p53 in non-small cell lung cancer (NSCLC) [68], and enhanced etoposide sensitivity in part by activating E2F1 and its downstream pro-apoptotic transcriptional programs [70]. TRIM28 has also been implicated in cisplatin resistance in NSCLC via transcriptional regulation of miR-125b-5p and the miR-125b-5p/cyclic AMP-responsive element-binding protein 1 (CREB1) axis [69]. In thyroid cancer, TRIM28 was shown to interact with P68/DEAD box protein 5 (DDX5) and positively regulate DDX5 expression at both the mRNA and protein levels, thereby supporting Wnt/β-catenin signaling [72].

Nevertheless, TRIM28 should not be defined as a uniformly anti-apoptotic factor, because its effect on apoptosis is context-dependent. In melanoma, for example, TRIM28 contributed to poly(I:C)-induced death receptor-associated apoptosis by mediating ubiquitin carboxyl-terminal hydrolase 27 (Usp27x)-dependent downregulation of cellular FLICE-like inhibitory protein long isoform (c-FLIP_L_) [73]. This example indicates that TRIM28 can either suppress or promote apoptotic susceptibility depending on the upstream stimulus, interacting partners, and dominant substrate network.

### 4.2. TRIM28 in Non-Malignant Apoptotic Stress Responses

Outside cancer, TRIM28 also shapes apoptotic outcomes in hematopoietic and stress-injury settings (Table 2). In knockout mouse models, erythroid-specific deletion of TRIM28 (*EpoR-Cre*, *Trim28^fl/fl^*) impaired adult erythropoiesis and caused anemia. Mechanistically, TRIM28 maintained erythrocyte transcriptional programs and heme synthetase gene expression; disruption of this program in TRIM28-deficient cells was accompanied by increased apoptosis in immature erythroid precursors [66]. Likewise, hematopoietic stem cell (HSC)-specific TRIM28 knockout (*Tie-Cre*, *Trim28^fl/fl^*) increased HSC division and apoptosis, promoted HSC egression from the bone marrow, and resulted in rapid HSC depletion, with effects linked to TRIM28-heterochromatin protein 1 (HP1) interaction [67]. In myocardial ischemia/reperfusion injury, TRIM28 promoted reactive oxygen species (ROS) accumulation and cardiomyocyte apoptosis by destabilizing glutathione peroxidase 1 (GPX1) through ubiquitination [64]. A recent report also indicated TRIM28 was involved in liquid–liquid phase separation (LLPS) events. Mechanistically, TRIM28-mediated SUMOylation of Ras GTPase-activating protein-binding protein 1/2 (G3BP1/2) regulated stress granule dynamics while modulating cellular ROS levels and reducing apoptosis [63]. In addition, stress-adaptive TRIM28 modification has also been implicated in apoptosis regulation in degenerative and injury-related settings. In intervertebral disk degeneration (IVDD), circGPATCH2L acted as a TRIM28 “decoy”, disrupting TRIM28 S824 phosphorylation, preventing p53 degradation, and thereby increasing apoptosis [74]. In stroke-related studies, phospho-TRIM28 (S824) stabilized proliferating cell nuclear antigen (PCNA) by antagonizing cullin 4A (CUL4A)-mediated ubiquitination, reducing apoptosis, and supporting neural stem cell survival after ischemia/reperfusion [65].

### 4.3. TRIM28 in Ferroptosis

Beyond apoptosis, multiple studies now place TRIM28 in ferroptosis regulatory circuitry (Figure 2 and Table 3), particularly through pathways linked to lipid metabolism and peroxidation, and iron homeostasis. In p53-wild-type hepatocellular carcinoma, unconventional prefoldin RPB5 interactor (URI) physically engaged TRIM28 and promoted TRIM28/MDM2-dependent p53 ubiquitination and degradation, thereby reprogramming lipid metabolism and buffering Tyrosine Kinase Inhibitor (TKI)-induced ferroptosis [75]. In spinal cord injury, TRIM28 promoted neuronal ferroptosis by binding acyl-CoA synthetase long-chain family member 4 (ACSL4), enhancing ACSL4 SUMO3 modification at lysine 532 (K532), and inhibiting K63-linked ubiquitination of ACSL4. This prevented optineurin (OPTN)-dependent selective autophagic degradation of ACSL4, thereby stabilizing ACSL4, increasing lipid peroxidation capacity, and amplifying ferroptotic stress [43]. By contrast, in injured cardiomyocytes, TRIM28 exerted a cardioprotective effect by acting as an E3 ubiquitin ligase for iron response element-binding protein 2 (IRP2). Mechanistically, TRIM28 promoted K48-linked ubiquitination of IRP2 at lysine 877 (K877), leading to IRP2 degradation, reduced transferrin receptor protein 1 (TFR1)-dependent iron uptake, and subsequent attenuation of iron overload, lipid peroxidation, and ferroptosis [40].

Conceptually bridging TRIM28’s chromatin role with ferroptosis susceptibility, the recently reported lipid oxygen radical defense pathway is epigenetically repressed by a complex containing zinc finger protein 354A (ZNF354A)-TRIM28-SET domain bifurcated histone lysine methyltransferase 1 (SETDB1)-activating transcription factor 2 (ATF2); oxidative lipid stress triggered phosphorylation-dependent complex disassembly, activating protective genes and altering ferroptosis sensitivity [20]. In neuroinflammatory settings, TRIM28 has also been reported to promote microglial ferroptosis by suppressing GSK3B expression and attenuating autophagy, thereby disturbing iron homeostasis, increasing oxidative stress, and enhancing pro-inflammatory cytokine release in neuropathic pain models [76].

These findings suggest that TRIM28’s net effect on ferroptosis depends on which ferroptosis module is dominant in a given tissue and stress setting: ACSL4-driven lipid remodeling in neuronal injury versus IRP2/TFR1-dependent iron handling in cardiomyocytes. Additional factors such as p53 status, basal iron metabolism, antioxidant capacity, oxidative stress intensity, inflammatory priming, and metabolic microenvironment may further determine whether TRIM28 raises or lowers the ferroptosis threshold. In addition to these more direct ferroptosis-linked mechanisms, TRIM28 may also influence ferroptosis susceptibility through regulation of ROS. These observations support a potential role for TRIM28 in ferroptosis-relevant ROS control, although this connection remains to be directly established through assessment of canonical ferroptosis markers and rescue with ferroptosis inhibitors.

### 4.4. TRIM28 in Autophagy, Mitophagy, and Proteostasis-Associated Survival

TRIM28 also intersects with autophagy programs, although current evidence suggests that this regulation more often shapes cell survival and proliferation than bona fide autophagy-dependent cell death. In glioblastoma, TRIM28 expression correlates with tumor grade and autophagy activity, and TRIM28 depletion suppresses autophagy and reduces tumor cell proliferation [78]. By contrast, in renal cancer cells, TRIM28 was reported to inhibit autophagy through a TRIM28-transcription factor E3 (TFE3)-lysine-specific demethylase 6A (KDM6A) transcriptional network, thereby restraining tumor cell proliferation [79]. Together, these studies indicate that TRIM28 can regulate autophagy, but the reported functional consequences are context-dependent and more closely linked to tumor growth and stress adaptation.

Evidence for a role of TRIM28 in mitophagy is emerging, but direct links to mitophagy-driven cell death outcomes remain limited. During erythropoiesis, TRIM28 regulates stage-specific mitophagy through a TRIM28-miRNA transcriptional cascade, thereby contributing to mitochondrial clearance during differentiation [80]. In host–pathogen interactions, TRIM28-dependent mitophagy has also been implicated in suppression of antiviral Janus kinase (JAK)-signal transducer and activator of transcription 1 (STAT1) signaling and facilitation of porcine epidemic diarrhea virus replication [81]. Phosphorylation of TRIM28 at S473 promotes the binding of TRIM28 to CBP80/20-dependent translation initiation factor (CTIF), inhibiting aggresome formation and thereby restricting viral proliferation [82]. TRIM28 has also been identified as an E3 ubiquitin ligase for Bcl-2-related protein A1 (BCL2A1), supporting a role in mitochondrial survival signaling [83]. Overall, these studies place TRIM28 in mitochondrial quality control, but more direct evidence will be needed to establish whether and how TRIM28-driven mitophagy regulates cell death outcomes.

### 4.5. TRIM28 in Inflammation-Associated Cell Death Signaling

Direct links between TRIM28 and canonical inflammasome-driven pyroptosis or canonical necroptosis remain relatively limited. Nevertheless, emerging studies suggest that TRIM28 can shape inflammatory pathways that intersect with these death programs. In a recent study, virus-triggered GSDMD C-terminal fragment (GSDMD-CT) engaged TRIM28 to promote selective autophagic degradation of retinoic acid-inducible gene I (RIG-I) and TANK-binding kinase 1 (TBK1). Mechanistically, TRIM28 mediated K48-linked polyubiquitination of RIG-I at lysine 181 (K181) and K27-linked polyubiquitination of TBK1 at lysine 487 (K487), which were recognized by the cargo receptors NDP52 and TOLLIP, respectively, thereby restraining type I interferon signaling [84]. TRIM28 has also been reported to enhance signal transducer and activator of transcription 3 (STAT3) transcriptional activity in B-cell chronic lymphocytic leukemia through cooperation with GM-CSFRα [85]. In addition, TRIM28 can influence inflammatory tissue injury outcomes that resemble regulated necrosis. In acute pancreatitis, the TRIM28-miR133a-cluster of differentiation 47 (CD47) axis impairs efferocytosis and potentiates pancreatic necrosis, although its direct role in necroptotic execution remains unclear [77]. During necroptosis, TRIM28 interacts with MLKL and undergoes Ser473 phosphorylation downstream of MLKL oligomerization through p38 MAPK activation, thereby promoting necroptosis-induced cytokine production. This places TRIM28 downstream of the MLKL execution step as an inflammatory-output regulator, rather than as an upstream activator of MLKL or a terminal necroptotic effector [30].

Together, these findings suggest that TRIM28 can influence inflammatory signaling and death-associated outputs at multiple levels, including antiviral signaling, cytokine production, efferocytosis, and regulated-necrosis-associated tissue injury. These examples also support a substrate-centered view of TRIM28-mediated cell death regulation: death modality is not dictated by ubiquitin linkage type alone; rather, linkage type determines the biochemical consequence for a given substrate, while the substrate’s pathway position and cellular context determine the death-related output. A key next step for the field is to determine when TRIM28 acts as a driver versus a modifier of cell death, and whether its chromatin/transcription and SUMO/ubiquitin-dependent functions coordinate coupled death programs under complex stresses (Figure 2).

## 5. TRIM33 and Cell Death Programs

**Figure 3:**
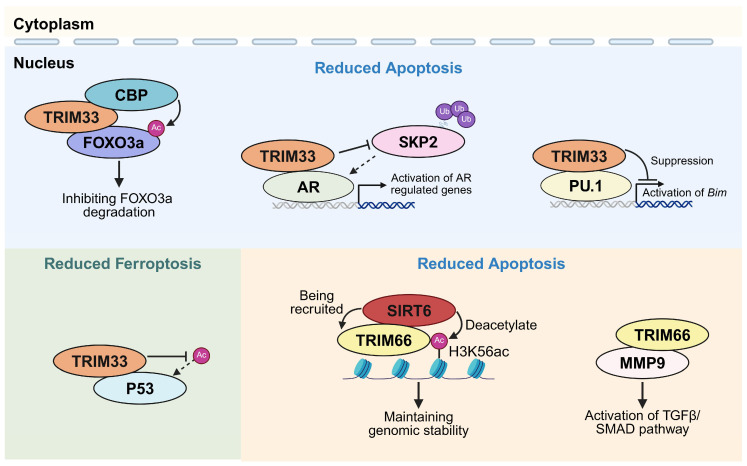
Representative mechanisms by which TRIM33 and TRIM66 regulate apoptosis and ferroptosis. This schematic illustrates several known mechanisms by which TRIM33 or TRIM66 modulates distinct cell death programs in different biological contexts. Ub, ubiquitin. Ac, acetylation. Created in BioRender. Yang, D. (2026) https://BioRender.com/rtgdns5 (accessed on 10 May 2026).

TRIM33 (TIF1γ) appears to influence cell death mainly by regulating transcription during differentiation or stress adaptation. Across the current literature, apoptosis is the most frequently reported death outcome associated with TRIM33, while ferroptosis has recently emerged as a TRIM33-regulated phenotype (Figure 3 and Table 4).

Early developmental work reveals that TRIM33 is required to maintain lineage programs and prevent apoptosis during hematopoiesis. In the zebrafish moonshine (mon) model, mon encodes the ortholog of mammalian TRIM33; hematopoietic progenitors in mon mutants fail to express normal levels of hematopoietic transcription factors, including gata1, and undergo apoptosis [89]. Similarly, TRIM33 deficiency increases apoptosis during early mouse embryoid body differentiation, accompanied by reduced Ras-related C3 botulinum toxin substrate 1 (RAC1) expression, a key survival factor [90].

Mechanistically, one of the better-resolved apoptosis-related mechanisms places TRIM33 at the enhancer level as a gatekeeper of pro-apoptotic gene activation. In B lymphoblastic leukemia, TRIM33 prevents apoptosis by silencing a purine-rich box-1 (PU.1)/TRIM33-co-occupied enhancer upstream of the pro-apoptotic gene Bcl-2 interacting mediator of cell death (*BIM*), thereby limiting enhancer-mediated *BIM* expression and sustaining leukemia cell survival; deletion of this enhancer renders TRIM33 dispensable for survival [22]. Another study reports that TRIM33 restrains apoptosis during dendritic cell development and maintenance, at least in part by suppressing PU.1-driven *BIM* transcription [91]. Several reports also connect TRIM33 to transforming growth factor-β (TGF-β) signaling, a pathway with highly context-dependent effects on differentiation, apoptosis, epithelial–mesenchymal transition, fibrosis, and metastasis. TRIM33 appears to reshape TGF-β/SMAD-dependent transcriptional output according to cell lineage, SMAD complex composition, chromatin state, disease stage, and microenvironmental cues. For example, TRIM33 has been identified as a chromatin reader and regulator of TGF-β signaling that cooperates with SMAD family member 4 (SMAD4) during palatogenesis [92], while downregulation of TRIM33 promotes TGF-β/SMAD signaling in clear cell renal cell carcinoma [93]. Thus, TRIM33-TGF-β crosstalk may influence cell death thresholds indirectly by altering stress-adaptive or pro-fibrotic transcriptional programs, although direct evidence linking this axis to a specific death modality in specific settings remains limited.

TRIM33 also modulates apoptosis under oxidative stress. In osteoblasts, TRIM33 overexpression restrained H_2_O_2_-induced apoptosis. The proposed mechanism involves TRIM33 interacting with CREB-binding protein (CBP) to limit CBP-mediated forkhead box O3 (FOXO3a) acetylation and subsequent ubiquitination/degradation, thereby preserving FOXO3a-dependent stress resistance [86]. In prostate cancer, TRIM33 is essential for tumor growth by reducing cell-cycle arrest and apoptosis, through facilitating androgen receptor (AR) chromatin binding and stabilizing AR against S-phase kinase-associated protein 2 (SKP2)-mediated degradation [87]. In a recent sepsis study, parthenolide was reported to functionally target TRIM33, suppress nuclear factor kappa-B (NF-κB) signaling, and reduce ubiquitin-dependent degradation of SMAD4, thereby exerting anti-inflammatory effects [94]; this finding raises the possibility that TRIM33 may also influence immune cell apoptosis, which requires further direct validation.

Ferroptosis represents a newer but notable TRIM33 axis. In hepatocellular carcinoma, TRIM33 has been reported to suppress tumor growth and metastasis while increasing ferroptosis susceptibility through E3 ligase-dependent regulation of transferrin receptor protein 1 (TFRC), linking TRIM33 to iron-handling control in ferroptosis [44]. By contrast, in osteoarthritis, TRIM33 downregulation was associated with increased p53 acetylation/stability and enhanced ferroptosis, thereby exacerbating disease progression [88], with direct TRIM33-dependent p53 acetylation-site evidence remaining limited. Together, these studies suggest that TRIM33 can influence ferroptosis through distinct upstream nodes, but additional work will be needed to define linkage type, substrate residues, and causal rescue mechanisms in each context.

## 6. TRIM66 and Cell Death Programs

Available studies suggest that TRIM66 (TIF1δ) primarily functions as a chromatin “reader” in the DNA-damage response (DDR) and a tumor-associated transcriptional/epigenetic regulator that supports stress adaptation. Current evidence places TRIM66 mainly at the chromatin-reader layer, where it may shape the repair and transcriptional context in which genotoxic stress is resolved or converted into apoptosis. Apoptosis is currently the most reproducible cell death phenotype observed after TRIM66 perturbation, particularly in cancer models, while evidence for other regulated cell death modalities remains sparse and largely indirect (Figure 3 and Table 5).

As a chromatin “reader” in DDR, TRIM66 reads unmodified H3R2-H3K4 via its PHD domain and H3K56ac via its bromodomain, thereby promoting genome stability in embryonic stem cells; loss of TRIM66 leads to elevated DNA damage and genomic instability, consistent with a role in buffering genotoxic stress [23]. In tumors, TRIM66 is frequently upregulated and is often associated with pro-survival phenotypes. In osteosarcoma, TRIM66 knockdown induces apoptosis and suppresses malignant phenotypes linked to epithelial–mesenchymal transition (EMT) and TGF-β signaling [95]. In triple-negative breast cancer cells (MDA-MB-468), TRIM66 silencing promotes apoptosis while suppressing epidermal growth factor receptor (EGFR) signaling and downstream JAK2/STAT3 activation [98]. Similarly, in NSCLC, TRIM66 silencing induced apoptosis and disrupted a matrix metallopeptidase 9 (MMP9)-TGF-β/SMAD module associated with malignant progression [99], whereas in glioma TRIM66 overexpression supports proliferation and metabolic adaptation via a cMyc-glucose transporter 3 (GLUT3) axis and limits temozolomide-induced apoptosis [97].

Consistent with a role in setting stress-adaptive survival thresholds, several studies further link TRIM66 to drug resistance programs, where apoptosis emerges when the axis is disrupted. In NSCLC, a lncRNA TATDN1/miR-451/TRIM66 axis has been reported to promote cisplatin tolerance [100]. Similarly, circSATB2/miR-150-5p/TRIM66 in NSCLC and circ_0051079/miR-625-5p/TRIM66 in osteosarcoma also converge on TRIM66 or its upstream ceRNA driver to regulate apoptosis and drug sensitivity [101,102]. In castration-resistant prostate cancer, a study of Zhoushi Qi Ling decoction proposed an SNHG10/miR-1271-5p/TRIM66 pathway linked to glycolysis-associated docetaxel resistance [103].

By contrast, evidence connecting TRIM66 to non-apoptotic cell death modalities remains limited. A ferroptosis-related lncRNA signature study in hepatocellular carcinoma noted TRIM66 among genes with differential mutation frequencies across risk groups, but this represents associative, model-derived linkage rather than mechanistic ferroptosis control [104]. Likewise, although TRIM66 repeatedly lies upstream of EGFR–JAK/STAT and TGF-β/SMAD pathways that can intersect with the inflammatory injury signaling, the current literature does not yet provide execution-level involvement in pyroptotic or necroptotic mechanisms.

Overall, available studies suggest that TRIM66 mainly influences apoptosis susceptibility indirectly by shaping chromatin-associated DNA damage responses, stress-adaptive transcriptional programs, and oncogenic survival pathways. Unlike canonical DDR proteins such as ATM or p53, TRIM66 is best interpreted as a chromatin-state reader and repair-context regulator rather than a primary damage sensor, kinase transducer, apoptotic transcriptional effector, or direct cell death executioner. Whether TRIM66 also plays a more direct role in other regulated cell death pathways remains to be determined.

## 7. TIF1 Proteins in Non-Malignant Stress Contexts

A major limitation of the current literature is that most mechanistic evidence linking TIF1 family proteins to regulated cell death comes from transformed or cancer cell models. This is important because the logic of cell death regulation in malignant cells may differ substantially from that in developmental, metabolic, ischemic, neurodegenerative, or fibrotic disease contexts.

### 7.1. Emerging Non-Malignant Stress Contexts Beyond Cancer

During recent years, TIF1-related cell death mechanisms in non-malignant stress contexts have begun to emerge. In developmental and hematopoietic systems, TRIM28 and TRIM33 are important for maintaining lineage-specific transcriptional programs and cell survival. Loss of TRIM28 in erythroid cells or hematopoietic stem cells increases apoptosis and disrupts tissue homeostasis [66,67], whereas TRIM33 deficiency in developmental hematopoietic models also leads to impaired lineage programming and apoptosis [89]. In parallel, TRIM66’s PHD–bromodomain cassette recognizes unmodified H3R2/H3K4 and H3K56ac and supports DNA damage responses and genome stability in embryonic stem cells [23].

In injury models, TRIM28 has been linked to apoptosis regulation in myocardial ischemia/reperfusion injury [64], stroke-related neural stem cell survival [65], and intervertebral disk degeneration [74]. It has also been implicated in ferroptosis regulation in non-malignant injury models: in spinal cord injury, TRIM28 promotes neuronal ferroptosis by binding ACSL4, enhancing ACSL4 SUMO3 modification at K532, and inhibiting K63-linked ACSL4 ubiquitination, thereby preventing OPTN-dependent autophagic degradation of ACSL4 [43]; in myocardial ischemia–reperfusion injury, TRIM28 acts instead as an E3 ligase for IRP2, promoting K48-linked ubiquitination of IRP2 at K877, reducing TFR1-dependent iron uptake, and suppressing cardiomyocyte ferroptosis [40]. These studies indicate that TIF1 proteins can regulate tissue injury through substrate- and context-dependent death-threshold mechanisms outside cancer.

In inflammatory diseases, TRIM24 and TRIM33 also show emerging relevance. TRIM24 restrains NLRP3/caspase-1/IL-1β-associated pyroptosis in endometriosis and osteoarthritis [47,54], whereas TRIM33 has been linked to NF-κB/SMAD-related inflammatory responses [94]. Together, these examples suggest that non-malignant TIF1 biology extends beyond tumor survival and involves tissue stress adaptation, repair, and regulated cell death susceptibility. Nevertheless, this evidence remains uneven across family members and disease systems, making tissue-specific validation essential before mechanisms derived from cancer models are applied to non-malignant diseases.

### 7.2. TIF1 Proteins in Kidney Injury and Kidney Disease

Kidney injury and disease are a particularly informative setting in which to consider TIF1 family biology, because renal injury is shaped not only by cell death itself, but also by how epithelial and stromal cells interpret stress, maintain lineage programs, and enter either adaptive repair or maladaptive fibrosis. Recent reviews emphasize that acute kidney injury (AKI), chronic kidney disease (CKD), and diabetic kidney disease (DKD) involve multiple interconnected death and stress-response pathways, including apoptosis, ferroptosis, pyroptosis, necroptosis, and repair-associated programs, rather than a single dominant mechanism [105,106].

Among the TIF1 family members, TRIM33 currently has the strongest direct link to non-malignant kidney disease. The clearest evidence comes from a tubular cell-specific deletion model, in which loss of TRIM33 exacerbated kidney injury-induced tubular polyploidy and fibrosis. Mechanistically, tubular TRIM33 was shown to suppress profibrotic EGFR and TGF-β signaling, and pharmacologic EGFR inhibition attenuated the worsened phenotype, supporting a protective role for TRIM33 in maladaptive repair during the AKI-to-CKD transition. The same study further reported reduced tubular TRIM33 expression in several CKD settings, including diabetic, hypertensive, and IgA nephropathy-associated human kidney disease [41]. More recent work also suggests a protective role for TRIM33 in diabetic renal injury: in high-glucose-treated mesangial cells, TRIM33 overexpression inhibited cell growth and fibrosis-related changes, consistent with the idea that restoring TRIM33 activity may restrain profibrotic signaling in diabetic kidney disease [107].

By contrast, current renal evidence for TRIM24 and TRIM28 is weighted more toward renal cancer than toward non-malignant kidney injury. In renal cell carcinoma (RCC), TRIM24 has been described as an oncogenic factor that promotes EMT and tumor progression [108]. Recent work further suggests that artesunate can enhance sunitinib sensitivity in RCC cells by upregulating TRIM24 and facilitating downstream ubiquitination events [109]. TRIM28 also shows renal relevance: TRIM28 has been reported to repress RCC cell proliferation by inhibiting TFE3/KDM6A-regulated autophagy [79]. These observations suggest that TRIM24 and TRIM28 are clearly relevant to renal disease biology, but at present their best-supported roles are in renal tumor adaptation, drug response, and tumor cell fitness rather than in primary AKI or CKD pathogenesis. In contrast, kidney-specific mechanistic evidence for TRIM66 remains sparse, and its role in renal injury or repair is still largely undefined. Overall, the available evidence suggests that the kidney is an interesting but still underdeveloped context for TIF1 family research: much more work will be needed to define how each TIF1 protein regulates renal cell death or shapes renal injury by controlling stress adaptation and repair processes.

## 8. Pharmacologically Targeting TIF1 Family Proteins

**Figure 4:**
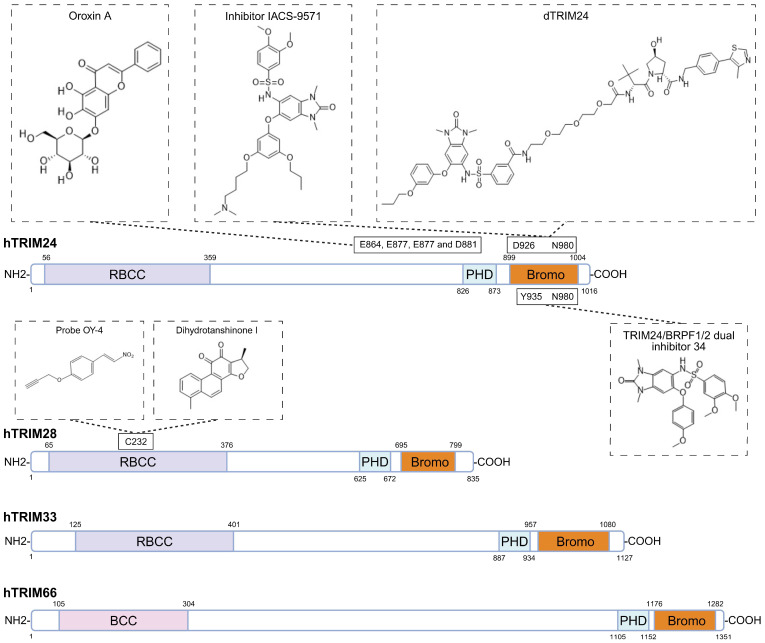
Structural organization of TIF1 family proteins with annotated binding sites for representative compounds. Schematic illustration of the domain structure of human TIF1 family proteins. Labeled are the known or putative hotspots bound by representative compounds reported to target each protein. Chemical structures are shown for the compounds discussed in the text. Because experimentally resolved ligand-TIF1 structural information is not currently available for all reported compounds, this figure is intended to summarize representative chemical matter. Reported structural information includes IACS-9571 bound to TRIM24 PHD–bromodomain (PDB: 4YAB) [18]. For TRIM28, docking-based studies have used available TRIM28 structural information (PDB: 8CR0) [111], but experimentally resolved ligand-TRIM28 co-crystal structures for these compounds are not yet available. RBCC, RING finger-B-box-coiled-coil domain; PHD, plant homeodomain finger; Bromo, bromodomain. Created in BioRender. Yang, D. (2026) https://BioRender.com/din86l1 (accessed on 10 May 2026).

From a drug-development perspective, TRIM24 is the most extensively studied therapeutic target within the TIF1 family, with a set of pharmacologic strategies reported, including the selective bromodomain chemical probe IACS-9571, the proteolysis targeting chimera (PROTAC) degrader dTRIM24, and natural compounds such as Oroxin A [18,19,46] (Figure 4). Early studies established that the TRIM24 bromodomain is chemically tractable, providing proof that TRIM24 can be directly targeted with small molecules. A benzimidazolone-based bromodomain inhibitor was reported in 2016 as an initial lead compound, with good selectivity and a K_D_ (dissociation constant) of 222 nM [110]. The same year, structure-guided optimization of N,N-dimethylbenzimidazolone bromodomain inhibitors, supported by iterative X-ray cocrystal analysis, led to the development of IACS-9571, a more potent and selective compound with higher affinity for TRIM24 (K_D_ = 31 nM) and favorable cellular and pharmacokinetic properties [18]. With an EC50 of 50 nM and an oral bioavailability of 29% [18], IACS-9571 emerged as a high-quality chemical probe for studying TRIM24 bromodomain function in vitro and in vivo. However, bromodomain homology remains a major limitation. IACS-9571 was developed through structure-guided optimization and shows high affinity for TRIM24, but it also binds BRPF1 with comparable low-nanomolar activity and should therefore be viewed more accurately as a dual TRIM24/BRPF1 chemical probe rather than a TRIM24-specific therapeutic candidate. Thus, although IACS-9571 has favorable chemical-probe properties, whether its selectivity window is sufficient for safe and durable in vivo therapeutic application remains unresolved.

Importantly, later work showed that pharmacologic degradation of TRIM24 can be more effective than bromodomain inhibition alone. Using the PROTAC degrader dTRIM24, Gechijian and colleagues demonstrated that targeted degradation of TRIM24 produced stronger antiproliferative effects than IACS-9571 in leukemia models, accompanied by apoptosis-associated readouts including poly ADP-ribose polymerase (PARP) cleavage and an increased sub-G1 fraction [19]. More recent work has further extended the therapeutic relevance of TRIM24 targeting. In BTZ-resistant mantle cell lymphoma, dTRIM24 not only induced apoptosis as a single agent but also restored BTZ sensitivity and markedly increased apoptotic cell death in BTZ-resistant cells, highlighting dTRIM24’s value as a strategy to re-engage BTZ-resistant cells’ death programs [55]. In parallel, Oroxin A has been reported as a TRIM24-targeting compound that promotes ferroptosis and suppresses colorectal tumor growth with limited toxicity, suggesting that TRIM24-directed strategies may engage not only apoptotic programs, but also non-apoptotic cell death pathways [46]. Together, the available studies support a TRIM24-targeting framework, spanning bromodomain inhibition, targeted degradation, and emerging ferroptosis-related therapeutic compound candidates.

Compared with TRIM24, TRIM28 remains a mechanistically attractive but pharmacologically less mature target. The earliest research targeting TRIM28 came from high-throughput screening for compounds that disrupt the MAGE-TRIM28 interaction, with the aim of relieving p53 suppression and inhibiting tumor growth [112]. Although this work identified several candidate hits, it did not yield pharmacological agents that progressed into broader translational development. More recently, alternative targeting strategies have begun to emerge. An anti-TRIM28 nanobody, NB237, reduced glioblastoma stem cell invasiveness and limited tumor spread in zebrafish models [113]. In addition, structure-based virtual screening identified Eltrombopag as a candidate TRIM28 inhibitor, and Eltrombopag treatment enhanced antitumor immunity and improved efficacy of anti-PD-1 therapy in clear cell renal cell carcinoma [114]. Activity-based protein profiling identified TRIM28 as a direct molecular target of dihydrotanshinone I (DHT), while docking analyses further suggested Cys232 of TRIM28 as a key binding site (Figure 4). DHT treatment reduced cisplatin-induced ROS accumulation and apoptotic signaling in a TRIM28-dependent manner in renal tubular cells [111]. Interestingly, a separate study focusing on PROTAC-based bromodomain-containing protein 4 (BRD4) degrader development also identified Cys232 of TRIM28 as a ligandable site engaged by the probe OY4 (Figure 4), further supporting the druggability of this residue [115]. Collectively, these studies suggest that pharmacological targeting of TRIM28 remains at an early stage. Meanwhile, TRIM28 functions as a major scaffold for KRAB-ZFP-mediated transcriptional repression and heterochromatin maintenance, including complexes involving HP1, SETDB1, NuRD-associated factors, and related chromatin-silencing machinery [116,117]. As a result, broad inhibition of TRIM28 protein–protein interactions could disrupt heterochromatin integrity, transposable element silencing, DNA damage responses, and genome stability. Future work will need to define direct target engagement, binding specificity, structural basis, functional selectivity, and safety for TRIM28-based strategies.

For TRIM33 and TRIM66, the pharmacological landscape is even less developed. Although both proteins have important biological functions, neither currently has validated selective inhibitors, degraders, or well-established druggable pockets comparable to those available for TRIM24. TRIM66 is particularly challenging because it lacks a canonical RING domain and is mainly understood as a chromatin-associated factor involved in broad DNA damage responses and genome stability.

Taken together, work on TRIM24 provides the most concrete example of pharmacological progress within the TIF1 family, moving from early evidence of chemical tractability to the development of highly effective chemical probes and targeted degraders. These studies offer a framework for future therapeutic exploration of other TIF1 family members. By contrast, although other TIF1 proteins, particularly TRIM28, show mechanistic and translational promise, their pharmacological development remains at a much earlier stage. TRIM28 has emerging targetability signals, but its central scaffold role in heterochromatin maintenance and genome stability creates substantial safety concerns. TRIM33 and TRIM66 currently lack effective chemical tools or validated druggable pockets.

## 9. Discussion and Future Perspectives

Current evidence suggests that TIF1 family proteins are regulators of cell death susceptibility rather than direct executors of death [13]. A common pattern across the TIF1 family is that cell death becomes evident when TIF1-dependent buffering is lost [22,23,42,61]. At the family level, apoptosis remains the most consistent and extensively studied phenotype, whereas links to ferroptosis, pyroptosis, and necroptosis are more selective and member-dependent [13,17].

Although TIF1 family proteins are frequently described to correlate with prosurvival or antiapoptotic effects, especially in cancer models, this likely reflects altered death susceptibility downstream of broader stress-regulatory functions rather than a universal anti-death identity. For example, TRIM28 often supports cell survival through transcriptional repression [59], DNA damage tolerance [29], p53 pathway modulation [60], proteostasis [43], or metabolic adaptation [75], but it can also promote apoptosis in specific settings, including through cFLIP_L_-related mechanisms [73]. TRIM33 provides another example of context-dependent and apparently contradictory biology. In TGF-β signaling, TRIM33 appears to reshape TGF-β/SMAD-dependent outputs according to tumor stage, cell lineage, SMAD complex composition, chromatin state, receptor/substrate turnover, and microenvironmental cues [41]. This interpretation is consistent with the broader dual role of TGF-β signaling, which can suppress early tumor growth but promote invasion, immune evasion, and metastasis in advanced disease. In some contexts, TRIM33 restrains tumor progression, such as by promoting nuclear β-catenin degradation, whereas in others, altered TRIM33 expression may modify TGF-β/SMAD-driven motility, EMT-like programs, or stress-adaptive responses. These contradictions are therefore not merely inconsistencies in the literature; they highlight the need to define TIF1 proteins as cell death susceptibility modulators whose effects depend on substrate networks, stress context, pathway priming, and microenvironment.

At the same time, the four family members are not positioned equally with respect to cell death. TRIM24 and TRIM28 are more often connected to death pathways in relatively direct ways [24,118,119], whereas TRIM33 and TRIM66 more often affect death indirectly by maintaining the cell state [22,23]. For TRIM24 and TRIM28, the literature repeatedly converges on nodes such as p53-dependent apoptosis, inflammatory signaling, redox stress, and ferroptosis-related pathways [42,60]. By contrast, TRIM33 and TRIM66 are more often involved in preserving differentiation programs, enhancer activity, genome stability, or tumor-adaptive transcription, so that apoptosis or ferroptosis-like phenotypes emerge when those programs fail [22,23]. This should not be taken as a difference in importance, but rather as a difference in how directly each protein engages pathways that trigger cell death.

Among the four family members, TRIM28 appears especially versatile because it has been studied most extensively and influences cellular fate through several interconnected layers [29,65]. First, TRIM28 can affect the stability and function of target proteins through ubiquitin- and SUMO-related regulation. Second, because TRIM28 lacks a sequence-specific DNA-binding domain, it most often acts as a transcriptional co-regulator that is recruited by KRAB zinc finger proteins or related partners, allowing it to reshape gene expression programs in a highly context-dependent manner. Third, TRIM28 is a core organizer of repressive chromatin states, in part through coupling to SETDB1-, HP1-, and H3K9me3-associated silencing complexes [116,117]. Finally, its activity can be rapidly redirected by stress-responsive post-translational phosphorylation, particularly at S473 and S824, which shift its downstream outputs under stress conditions [28,120]. Taken together, these properties place TRIM28 in a unique position to connect target-protein fate [40,43], chromatin accessibility and transcriptional control [20], and stimulus-responsive signaling [28], thereby shaping how cells adapt to stress and how cell death programs are engaged.

TIF1 proteins become especially interesting when cell death is not confined to a single pathway. In stressed or damaged tissues, different cell populations may undergo different forms of death in parallel, and in some settings, a single cell may engage mixed or coupled death responses rather than one pathway in isolation. This is where TIF1 proteins may be particularly informative: because they act upstream of execution and help shape transcriptional, inflammatory, and metabolic conditions, they are well placed to influence more integrated cell death outputs [67,121]. Although current evidence does not support TRIM24 or TRIM28 proteins physically associating with PANoptosome components or direct coordinators of mixed cell death programs, both proteins repeatedly intersect with determinants of apoptotic competence [42,60], inflammatory signaling [47,84], and stress integration [56,63]. This pattern suggests that TIF1 proteins may influence PANoptosis-relevant pathway priming or broader mixed-death susceptibility, but this remains to be tested directly. From a translational standpoint, this upstream position is attractive because it may provide leverage over maladaptive survival, therapy resistance, and inflammatory tissue injury, but it also raises obvious concerns about specificity and context-dependent effects [16,119].

Several questions now stand out. Meaningful family-wide comparisons are still lacking. Beyond apoptosis, the evidence remains uneven, and stronger execution-level data will be needed to define how TIF1 proteins intersect ferroptosis, pyroptosis, necroptosis, autophagy/mitophagy-associated stress responses, and PANoptosis-relevant circuitry. Finally, any future therapeutic strategy will need to balance sensitizing diseased cells against preserving normal developmental, hematopoietic, and neural homeostasis. A clearer picture of how TIF1 proteins connect stress signaling to specific cell death decisions may therefore open new opportunities to reprogram maladaptive survival across diseases, including cancer, inflammation, and tissue injury.

## 10. Conclusions

TIF1 family proteins occupy a distinctive position at the interface of chromatin regulation, post-translational modification, stress signaling, and cell-fate control. Rather than acting as canonical cell death executioners, they appear to tune the susceptibility of regulated cell death in a context-dependent manner. Therapeutically, TIF1 proteins offer intriguing opportunities to modulate maladaptive survival, therapy resistance, inflammatory injury, and failed tissue repair. However, these opportunities must be approached with caution because TIF1 proteins also support normal genome stability, chromatin organization, and stress adaptation. Future studies that define substrate-specific mechanisms, tissue-specific functions, and druggable vulnerabilities will be essential for translating TIF1 biology into safe and effective therapeutic strategies.

## Figures and Tables

**Table 1:** Representative studies supporting TRIM24 involvement in cell death regulation.

Disease/Model	Expression	Impact on Cell Death	Mechanism	Ref.
Human breast cancer cells	N/A	Suppress apoptosis	TRIM24 ubiquitylated and negatively regulated p53.	[42]
Lung squamous cell cancer	Elevated in NRF2-depleted cells	Suppress apoptosis and ferroptosis	TRIM24 sustained oxidative stress tolerance through TRIM24-PI3Kα complex formation, whereas TRIM24 loss destabilized PI3Kα	[45]
Colorectal cancer	Elevated	Suppress ferroptosis	TRIM24 may promote TSPO ubiquitination and limit TSPO accumulation. Pharmacologic downregulation of TRIM24 by Oroxin A reduced TSPO ubiquitination.	[46]
Endometriosis	Decreased	Suppress pyroptosis	TRIM24 interacted with NLRP3 and facilitated NLRP3 ubiquitination.	[47]
Acute myeloid leukemia	Elevated	Suppress apoptosis	TRIM24 modulated Wnt/GSK3β/β-catenin signaling.	[48]
Hepatocellular carcinoma	Elevated	Suppress apoptosis	TRIM24 promoted the DNA damage response by facilitating MRN complex recruitment to double-strand breaks	[21]

**Table 3:** Representative studies supporting TRIM28 involvement in ferroptosis, necrosis, and autophagy regulation.

Disease/Model	Expression	Impact on Cell Death	Mechanism	Ref
Hepatocellular carcinoma/TKI resistance	N/A	Suppress ferroptosis	TRIM28 interacted with URI and promoted p53 ubiquitination and degradation. p53, in turn, negatively regulated SCD1, which affected monounsaturated fatty acids generation.	[75]
Myocardial I/R injury	Decreased	Suppress ferroptosis	TRIM28 promoted the ubiquitination and degradation of IRP2, thereby inhibiting TFR1-mediated iron uptake and alleviating intracellular iron overload and lipid peroxidation.	[40]
Spinal cord injury	N/A	Increase ferroptosis	TRIM28 promoted SUMOylation of ACSL4 and suppressed autophagic degradation of ACSL4.	[43]
Cell lines	N/A	Suppress ferroptosis	An ATF2–SETDB1–TRIM28–ZNF354A epigenetic complex regulated antioxidant genes under oxidative stress.	[20]
Neuropathic pain and neuroinflammation	Elevated	Increase ferroptosis	TRIM28 modulated autophagy and iron homeostasis by suppressing GSK3B.	[76]
Acute pancreatitis	Elevated	Increase necrosis	TRIM28 positively regulated CD47 through miR133a. CD47 knockout mouse and anti-CD47 antibody treatment alleviated pancreatic necrosis.	[77]
Glioblastoma	Elevated	Increase autophagy	TRIM28 promoted autophagy-related 5 (ATG5) expression.	[78]

**Table 4:** Representative studies supporting TRIM33 involvement in cell death regulation.

Disease/Model	Expression	Impact on Cell Death	Mechanism	Ref
Osteoblasts in osteoporosis	Decreased	Suppress apoptosis	TRIM33 interacted with CBP to limit CBP-mediated FOXO3a acetylation and subsequent ubiquitination/degradation.	[86]
Prostate cancer	Elevated	Suppress apoptosis	TRIM33 stabilized the androgen receptor (AR) against SKP2-mediated degradation.	[87]
Osteoarthritis	Decreased	Suppress ferroptosis	TRIM33 limited p53 acetylation/stability.	[88]
Hepatocellular carcinoma	Decreased	Increase ferroptosis	TRIM33 enhanced the ubiquitination of TFRC.	[44]
B lymphoblastic leukemia	N/A	Suppress apoptosis	TRIM33 silenced a PU.1/TRIM33-co-occupied enhancer upstream of the pro-apoptotic gene *BIM* and suppressed *BIM* transcription.	[22]

**Table 5:** Representative studies supporting TRIM66 involvement in cell death regulation.

Disease/Model	Expression	Impact on Cell Death	Mechanism	Ref.
Osteosarcoma	Elevated	Suppress apoptosis	TRIM66 activated the TGF-β signaling pathway and inhibited P53 expression.	[95]
Colorectal cancer	Elevated	Suppress apoptosis	TRIM66 activated the JAK2/STAT3 signaling pathway.	[96]
Glioma	Elevated	Suppress apoptosis	TRIM66 increased glucose uptake and ATP production through positive regulation of c-MYC and GLUT3.	[97]
Breast cancer	Elevated	Suppress apoptosis	TRIM66 activated the EGFR signaling pathway.	[98]
NSCLC	Elevated	Suppress apoptosis	TRIM66 activated TGF-β/SMAD signaling pathway through binding with MMP9.	[99]

## Data Availability

No new data were created or analyzed in this study. Data sharing is not applicable to this article.
